# Editorial: Molecular innate immunity in aquatic animals and their response to epidemic diseases

**DOI:** 10.3389/fimmu.2025.1608192

**Published:** 2025-04-24

**Authors:** Hu Lu, Zhiwei Liao, Jiejie Sun, Linwei Yang, Hengwei Deng

**Affiliations:** ^1^ School of Marine Biology and Fisheries, Collaborative Innovation Center of Marine Science and Technology, Hainan University, Haikou, China; ^2^ Pelotonia Institute for Immuno-oncology, Comprehensive Cancer Center—James Cancer Hospital and Solove Research Institute, College of Medicine, The Ohio State University, Columbus, OH, United States; ^3^ Liaoning Key Laboratory of Marine Animal Immunology and Disease Control, Dalian Ocean University, Dalian, China; ^4^ State Key Laboratory of Mariculture Breeding, Key Laboratory of Marine Biotechnology of Fujian Province, College of Marine Sciences, Fujian Agriculture and Forestry University, Fuzhou, China

**Keywords:** innate immunity, aquatic animals, epidemic diseases, Aeromonas hydrophila, antibacterial immune

In early 2025, as an observer for the journal Frontiers in Immunology, I was honored to be invited to write an editorial on the immune mechanisms of aquatic animals. The editorial is primarily based on six significant research papers recently published in this Research Topic, which collectively reveal critical insights into the immune mechanisms of aquatic animals from multidisciplinary perspectives, providing new theoretical support and practical guidance for the sustainable development of the aquaculture industry.

*Aeromonas hydrophila*, a devastating bacterial pathogen in aquaculture, causes severe economic losses through motile aeromonas septicemia (MAS). Two research articles are focused on the innate immune response to *A. hydrophila* infection. An interleukin-22 (IL-22) was identified and characterized from *Megalobrama amblycephala*, revealing the cytokine’s triple regulatory role in alleviating *Aeromonas hydrophila*-induced damage through suppression of ROS/NLRP3 inflammasome activation. IL-22 simultaneously suppresses inflammation, enhances antioxidant defenses, and promotes tissue repair, establishing its potential for developing IL-22-based vaccines and selective breeding strategies. Eight dual-specificity phosphatase (DUSP) genes (pfDusp1–7 and pfDusp10) were highly expressed in the kidneys during *A. hydrophila* infection in *Pelteobagrus fulvidraco*, and precisely regulates the inflammatory response through the dephosphorylation of the MAPK pathway. This finding reveals the unique immune homeostasis maintenance mechanism of teleost fish.

Three other studies have explored the antibacterial immune mechanisms of aquatic animals in depth from different angles. He et al. investigated the molecular mechanisms by which acute hypoxia stress suppresses the antibacterial immunity of *Litopenaeus vannamei*, thereby increasing susceptibility to *Vibrio parahaemolyticus* infection. The study identified the HIF-1α-Yki-Cactus axis as a key regulator in hypoxia-induced immune suppression and established a link between environmental stress, metabolic reprogramming, and pathogen virulence. This mechanism provides new insights into the interactions between hypoxia, host immunity, and bacterial infections, offering a theoretical basis for optimizing aquaculture practices to mitigate disease outbreaks in hypoxic environments. Qin et al. investigated the role of Ring Finger Protein 5 (RNF5) in regulating the STING-mediated antiviral immune responses in *Siniperca chuatsi*. The study identified ubiquitination sites at K135/K155 on the ScSTING protein, revealing a novel viral defense mechanism absent in mammals. Targeting these sites could offer a potential breakthrough in the development of broad-spectrum antiviral drugs. In a separate study, Pan et al. identified and characterized 10 galectins (ToGals) in *Trachinotus ovatus* and analyzed their potential roles in immune responses. The research demonstrated that ToGals expression was modulated in response to infections by *Cryptocaryon irritans* and *Streptococcus agalactiae*, suggesting their involvement in immune defense mechanisms. In particular, the key member ToGal-3like dual localization in both the nucleus and cytoplasm, and demonstrated the ability to agglutinate various bacteria, suggesting its potential as a pattern recognition receptor (PRR). The study underscored the role of galectins as PRRs in teleosts and their role in innate immunity.

In addition, another significant study examined the effects of mycotoxins on the innate immune system of aquatic animals. Zheng et al. explored the immunotoxicity of ochratoxin A (OTA) in zebrafish, focusing on its impact on innate immunity. The study found that OTA exposure inhibited embryonic development, reduced neutrophil and macrophage numbers, and impaired immune cell migration during fin regeneration. Transcriptome analysis showed that OTA upregulated the apoptosis pathway mediated by anxa1a and anxa1d in neutrophils. Moreover, aesculetin was shown to mitigate the immunotoxic effects of OTA, indicating its potential as a therapeutic agent.

These studies have not only provided new theoretical foundations for aquatic animal immunology but also offered valuable guidance for aquaculture practices. Future research should integrate metabolomics with transcriptomic analyses to explore how metabolic reprogramming under stress conditions influences immune responses, potentially identifying novel targets for enhancing disease resistance. Comparative analyses of immune gene families across species are essential to uncover conserved and species-specific regulatory mechanisms, which could inform the development of targeted therapeutics for various aquaculture species. It is worth mentioning that the study of hypoxic stress inhibiting antibacterial immunity by disrupting the hepatopancreas structure and regulating the Yki-Cactus pathway has provided a new perspective for the prevention and control of the summer mortality syndrome in shrimp. The integration of environmental sensors with gene-editing technologies holds the potential to achieve a dual breakthrough in dynamic dissolved oxygen control and the breeding of disease-resistant varieties, promoting the development of the aquaculture industry toward greater intelligence and sustainability.

